# Observational study of clinical outcomes for testosterone treatment of pubertal delay in Duchenne muscular dystrophy

**DOI:** 10.1186/s12887-019-1503-x

**Published:** 2019-04-25

**Authors:** C. L. Wood, T. D Cheetham, K. G Hollingsworth, M. Guglieri, Y. Ailins-Sahun, S. Punniyakodi, A. Mayhew, V. Straub

**Affiliations:** 1 John Walton Muscular Dystrophy Research Centre, Institute of Genetic Medicine, Newcastle University, Newcastle, UK; 2 Institute of Genetic Medicine, Newcastle University and Great North Children’s Hospital, Royal Victoria Infirmary, Queen Victoria Road, Newcastle upon Tyne, UK; 3 Newcastle Magnetic Resonance Centre, Institute of Cellular Medicine, Newcastle University, Newcastle, UK; 4 Department of Paediatrics, Plymouth Hospitals NHS Trust, Plymouth, UK; 5 New Cross Hospital, Wolverhampton NHS Trust, Wolverhampton, UK

**Keywords:** Duchenne muscular dystrophy, Pubertal delay, Testosterone, Glucocorticoids

## Abstract

**Background:**

Adolescents with DMD treated with chronic high dose GC therapy typically have profound pubertal delay. Testosterone, the main circulating androgen in men, promotes virilisation and growth with associated accrual of fat-free muscle mass and bone mineral content. Testosterone therapy is routinely used to mimic the normal stages of pubertal development in patients with hypogonadotrophic hypogonadism, androgen deficiency secondary to testicular disease and in constitutional delay of growth and puberty (CDGP). Improved life expectancy in DMD has meant that more adolescents are eligible for testosterone supplementation but there is little objective data regarding the impact of this treatment on muscle structure and function, bone integrity and overall well-being.

**Methods:**

This is a single centre observational clinical trial (NCT02571205) that aims to follow the progress of 15 adolescents with Duchenne muscular dystrophy and delayed puberty as they are managed with incremental testosterone therapy to induce puberty. Subjects will all be treated with a steadily increasing dose of testosterone administered by injection every 4 weeks and data will be collected to help us determine the effectiveness and tolerability of the described treatment regimen. We will use the data to explore the effects of testosterone on pubertal development, growth, muscle strength and function, bone mineral density, body composition with a detailed record of any adverse events. We will also carry out interviews to explore the boys’ views on the tolerability of the regimen. The study will last for 27 months in total for each participant.

**Discussion:**

Our experience has indicated that testosterone treatment in adolescents with DMD is liked and well tolerated but we have not collected objective data on a specific treatment regimen and there is no current consensus. Testosterone supplementation is not part of the standard of care of pubertal delay in DMD but inclusion in future protocols may be appropriate depending on the results of this trial.

**Trial registration:**

EudraCT Number: 2015–003195-68. Research Registry & References: Clinical trials.gov- NCT02571205 (registered 8/10/15).

**Electronic supplementary material:**

The online version of this article (10.1186/s12887-019-1503-x) contains supplementary material, which is available to authorized users.

## Background

Duchenne muscular dystrophy (DMD) is a severe and ultimately fatal, X-linked genetic disease that affects up to 1 in 3500 live male births [1]. DMD occurs as a result of mutations in the gene at 21p on the X chromosome with an associated reduction or complete loss of dystrophin protein. DMD is the most common muscular dystrophy in childhood and is associated with progressive muscle weakness. Untreated individuals lose ambulation by a mean age of 9.5 years [2]. Respiratory, cardiac or orthopaedic complications usually cause premature death in the second or third decade [3]. Whilst there is still no cure for DMD, therapeutic advances such as the routine use of glucocorticoids (GC) and co-ordinated multidisciplinary care have dramatically improved the course of the disease [4]. Patients with DMD now regularly survive into their thirties [5]. GC are the key treatment in DMD and are the only medical therapy proven to stabilise muscle strength for a limited period [6]. Continuing longitudinal follow-up of steroid-treated cohorts has found that long-term, regular use of GC is associated with improved muscle strength and 3 additional years of independent ambulation with preserved upper limb function [7]. In addition GC improve and maintain respiratory function [8], delay the development of orthopaedic complications [9] (e.g. scoliosis) and may have a positive impact on cardiac function [10]. Unfortunately the use of GC in DMD is associated with frequent and substantial side-effects that are a major issue for many patients. A range of organ systems are affected with growth retardation [11], impaired bone mineralisation (increasing risk of vertebral and lower limb fractures [12]), impaired glucose metabolism, cataracts [13], adrenal suppression, and pubertal delay.

Pubertal delay is one of the main concerns reported by adolescents with DMD on long-term treatment with high dose GC and is an almost universal finding. GC inhibit the production of factors regulating the hypothalamic-pituitary axis during puberty resulting in hypogonadotrophic hypogonadism. One study showed that by 11.5 +/− 2.9 years of age, 97% of boys were pre-pubertal with an average height z-score of − 2.9 [14]. Similarly, 43/44 GC-treated boys in an American DMD cohort were pre-pubertal at 13 years of age or older [15]. Delayed puberty may have a number of detrimental effects. Adverse psychosocial and educational consequences of failure to advance through puberty are well recognised and adolescents with DMD get frustrated as people often think they are younger than they are. For some parents the psychosocial stress associated with the disorder and its treatment exceeds that caused by the physical aspects of the condition [16]. A study of subcutaneous testosterone therapy in 18 adolescents with primary hypogonadism demonstrated an improvement in psychosocial outlook and self-image [17]. This is consistent with reports of increased cheerfulness and relaxation in hypogonadal men receiving androgen replacement [18, 19]. In some studies the mean age of initiation of androgen therapy is well after a point at which normal pubertal development is typicallyy observed [20]. Pubertal delay is often associated with significant emotional distress and so this delay should be avoided.

The delayed puberty and lack of an associated pubertal growth spurt coupled with the short stature associated with DMD means that by adolescence almost all those with DMD are very short and look much younger than their age. As the standards of care have changed and health of adolescents with DMD has improved, the expectations of the patients have also changed with more young people seeking to establish relationships and to lead independent adult lives.

As a result, we face new challenges in the follow-up and supportive care of those with DMD. Families are increasingly seeking an endocrine specialist opinion to discuss possible treatment options for short stature and pubertal delay. This is not a straightforward consultation due to the likely multifactorial nature of the growth failure and pubertal delay and the need to consider concomitant GC use and potential side effects.

### Rationale for the study

Testosterone is the main circulating androgen in men and promotes virilisation and growth with associated accrual of fat-free muscle mass and bone mineral content. Testosterone levels of up to 30 times greater than those found during childhood are required to initiate and maintain puberty. Testosterone supplementation is routinely used to mimic the normal stages of pubertal development in patients with hypogonadotrophic hypogonadism, androgen deficiency secondary to testicular disease and in constitutional delay of growth and puberty (CDGP). However, DMD is a rare disease and testosterone therapy has only been given in recent years as life-expectancy has improved. There are very few data regarding the benefit of testosterone treatment in DMD, especially in relation to muscle and bone. A recent local audit of the 14 adolescents with DMD treated with testosterone for pubertal delay found that there was some height gain but the usual growth increment from puberty was compromised [21]. Few had adult levels of endogenous testosterone levels at the end of the study period despite being on treatment for over 4 years in some cases, suggesting that sub-optimal treatment regimens may have been used or that the hypothalamic-pituitary axis remains suppressed with ongoing GC use.

Testosterone is currently only offered to a minority of adolescents with DMD based on the experience and expertise of the clinician involved in patient care. Limited evidence suggests that testosterone is a useful and well-liked adjuvant therapy that can help address two of the boy’s main concerns which are short stature and pubertal delay. There is, therefore, a clear need for a study to systematically evaluate treatment satisfaction and efficacy of testosterone in DMD adolescents with pubertal delay. There is a need to confirm and evaluate the effect of testosterone on key outcomes such as testicular volume, bone strength, growth and muscle function. There is also lack of information regarding the impact of testosterone replacement therapy on overall quality of life in this population. It is becoming increasingly acknowledged that the patient’s experience of health is vitally important. We have decided to employ a mixed methods approach for the primary outcome measure. As well as routine clinical outcome measures, we will also carry out qualitative interviews on a subset of the population studied to gauge their ideas, concerns and expectations in more depth.

### Trial objectives

#### Primary

- Evaluate patient satisfaction in response to testosterone replacement therapy in patients with DMD and pubertal delay

#### Secondary

- Evaluate the effect of an incremental regimen of intramuscular testosterone therapy on pubertal staging, testicular volume and sex hormone levels
- Evaluate the effect of testosterone supplementation on growth
- Evaluate the effect of testosterone replacement therapy on muscle strength, mass and function
- Evaluate the effect of testosterone replacement therapy on muscle pathology as assessed by muscle MRI
- Evaluate the effect of testosterone replacement therapy on bone mineral density and body composition

## Methods

### Trial design and setting

This is a single-centre, prospective trial investigating the clinical outcomes of pubertal induction with an incremental regimen of intramuscular testosterone replacement therapy in adolescents with DMD and delayed puberty. The trial is being conducted at the Clinical Research Facility, Royal Victoria Infirmary in Newcastle upon Tyne, UK.

### Study population

Potential participants will be identified during routine follow-up appointments by clinicians from the John Walton Muscular Dystrophy Research Centre in Newcastle. Potentially eligible subjects and their parent(s)/guardian(s) (with legal authority to consent on behalf of the child) will be invited to attend an initial screening visit.

#### Eligibility criteria

### Inclusion criteria

- A molecular diagnosis of Duchenne Muscular Dystrophy.
- Males aged between 12 and 17 years of age at time of first dosing
- Pre-pubertal (Tanner stage 1, testicular volume < 4 mls, initial testosterone level of < 2.0 nmol/l)
- Subjects are receiving the standard of care for DMD as recommended by the NorthStar UK [22] and recently revised care guidelines [23].
- Patients are capable of sitting upright in a wheelchair for at least an hour
- Patients have stable respiratory function. Artificial ventilation with either Bipap/cPAP or tracheostomy is not a contraindication to the study.
- Informed consent/assent signed by the patient (or parent/guardian and assent if under 16 years of age)

### Exclusion criteria

- Severe learning difficulties that would preclude them from cooperating with examination.
- Anticipated surgery during the study period.
- Symptomatic cardiac failure.
- Participants/families who may have emotional or psychological problems if recruited to a study
- Hypersensitivity to the active substance or to any of the excipients, including arachis oil or derivatives (including hypersensitivity and allergy to peanuts or soya.)
- Any contra-indication to receiving an intramuscular injection
- Any additional chronic disease that affects androgen production
- Anti-coagulant therapy
- If participation in the study is not recommended in the opinion of the investigators

#### Intervention

Sustanon is widely used in the UK for androgen replacement therapy and is routinely used, by prescription, to treat pubertal delay. Sustanon 250 contains four esters of testosterone with different durations of action:

**Table:** 

1. Testosterone propionate	30 mg/ml
2. Testosterone phenylpropionate	60 mg/ml
3. Testosterone isocaproate	60 mg/ml
4. Testosterone decanoate	100 mg/ml

(Equivalent to a total of 176 mg of Testosterone)

The esters are hydrolysed into the natural hormone testosterone as soon as they enter the general circulation. Sustanon 250 contains the excipients 1 ml Arachis oil and benzyl alcohol. It is commercially available from Merck Sharp & Dohme Limited. An SmPC is available [24].

Sustanon 250 is administered by deep intramuscular injection. Patients will follow a standard dose incremental regimen for 2 years:

- Sustanon 50 mg (0.2 ml) every 4 weeks for 12 weeks
- Sustanon 100 mg (0.4 ml) every 4 weeks for 40 weeks
- Sustanon 150 mg (0.6 ml) every 4 weeks for 24 weeks
- Sustanon 250 mg (1 ml) every 4 weeks for 28 weeks

The first injection will be given to each subject in the Clinical Research Facility. Subsequent injections will either be given locally, at the subject’s General Practitioners, or by local endocrine nurses, according to individual preference and arrangements. A copy of the suggested regimen will be given to all practitioners who will be administering the testosterone; they will be asked to document any reason for deviating from this protocol on a form which is provided as part of the study documents. Sustanon will be drawn up according to the protocol and administered by a practitioner competent in giving intramuscular injections. The medication will be prescribed by a clinician according to the protocol, and dispensed to the patient according to local pharmacy policy.

The acute toxicity of Sustanon is low. If symptoms of chronic overdose occur (such as polycythaemia and priapism) then the drug should be discontinued or temporarily paused. The practitioner will be encouraged to discuss this with the Chief Investigator before deciding on the most appropriate course of action, or to record their action and reason for it. The study does not foresee any safety issues, as the patients will follow a standard incremental regimen and undergo assessments consistent with those used in routine clinical practice.

#### Assessment of compliance

Each participant will have an injection record to fill in the date and dose of each injection given. Where feasible, study visits will coincide with routine clinical follow-up, to enhance the likelihood of good compliance. Visit windows of +/− 14 days should ensure visit attendance; non-attendance for study visits will prompt follow-up by telephone by the study investigator.

## Discussion

### Study procedures

#### Baseline assessments & data

If a subject is confirmed to have pubertal delay at screening, he will be asked to return for a baseline visit. At the baseline visit, he will be enrolled in the study (Table 1). Only one patient failed screening, as they had recently stopped their GC and were already peri-pubertal.

**Table 1:** Schedule of Events

	Screening	Visit 1	Visit 2	Visit 3	Visit 4	Visit 5	Visit 6	Visit 7	Visit 8	Visit 9	Visit 10
		0 weeks (+/− 14 days)	12 weeks (+/− 14 days)	24–28 weeks (+/− 14 days)	40 weeks (+/− 14 days)	52 weeks (+/− 14 days)	64 weeks (+/− 14 days)	76–80 weeks (+/− 14 days)	92 weeks (+/− 14 days)	104 weeks (+/− 14 days)	116 weeks (+/− 14 days)
Clinic based evaluations											
Informed consent	X										
Inclusion criteria	X										
Exclusion criteria	X										
Demography	X										
Medical history	X	X									
Ability to comply with study evaluations	X										
Height	X	X	X	X	X	X	X	X	X	X	
Weight	X	X	X	X	X	X	X	X	X	X	
BP and other vital signs	X	X	X	X	X	X	X	X	X	X	
Skin examination		X	X	X	X	X	X	X	X	X	
Pubertal examination	X	X		X		X		X		X	
General physical examination		X		X		X		X		X	
Details of concomitant meds		X	X	X	X	X	X	X	X	X	
Details of Adverse Events		X	X	X	X	X	X	X	X	X	
Urine and blood tests											
Urine for bone turnover markers/steroid profile		X		X		X		X		X	
Blood for haematology/chemistry		X		X		X		X		X	
Blood for testosterone, LH, FSH levels	X	X	X	X	X	X	X	X	X	X	X
Blood for other hormone levels		X				X				X	
Blood for lipid profile		X				X				X	
Blood for 25-OH-D		X		X		X		X		X	
Blood for Ca/bone turnover markers		X				X				X	
Optional blood sample for Biobank		X				X				X	
Functional assessments											
Respiratory function (FVC)		X		X		X		X		X	
NSAA and/ or PUL		X		X		X		X		X	
Timed and graded functional tests		X		X		X		X		X	
Muscle strength/ROM		X		X		X		X		X	
6 MWT		X		X		X		X		X	
Questionnaire assessments											
TSQM		X		X		X		X		X	
PEDSQoL – parent		X		X		X		X		X	
PEDSQoL – child		X		X		X		X		X	
Semi-structured interview		X								X	
Investigations											
DXA		X				X				X	
Muscle MRI		X				X				X	
Echo		X				X				X	
ECG		X				X				X	
Wrist X-ray		X								X	

The following tests will be carried out:

- Physical examination and vital signs (including height, weight, blood pressure, pulse)
- Medical history (including current medications, vitamins and supplements)
- Blood sample for routine laboratory tests, blood hormone levels, vitamin D and bone markers.
- Urine sample collection for analysis of bone markers.
- Motor skills test (jumping, hopping, time to stand from lying)
- Standard assessments of muscle strength, function and range of movement
- The North Star Ambulatory Assessment including six-minute walk test (6MWT) if subject can walk. If subject is unable to walk unaided, he will be asked to do the Performance of the Upper Limb (PUL) instead
- Physiotherapy assessment and advice
- Lung capacity test.
- Full-body DXXA scan for bone and muscle mass (part of routine care)
- X-ray of wrist to give information on growth and physical development (part of routine endocrine care for delayed puberty)
- Heart function tests – electrocardiogram and echocardiogram (part of routine care)
- Muscle MRI of arm and legs.
- Quality of Life questionnaire completion (PedsQL questionnaires).
- Treatment Satisfaction for Medication questionnaire completion.
- Semi-structured interviews (approx. 1 h)

### Subsequent trial assessments

**Fig. 1:**
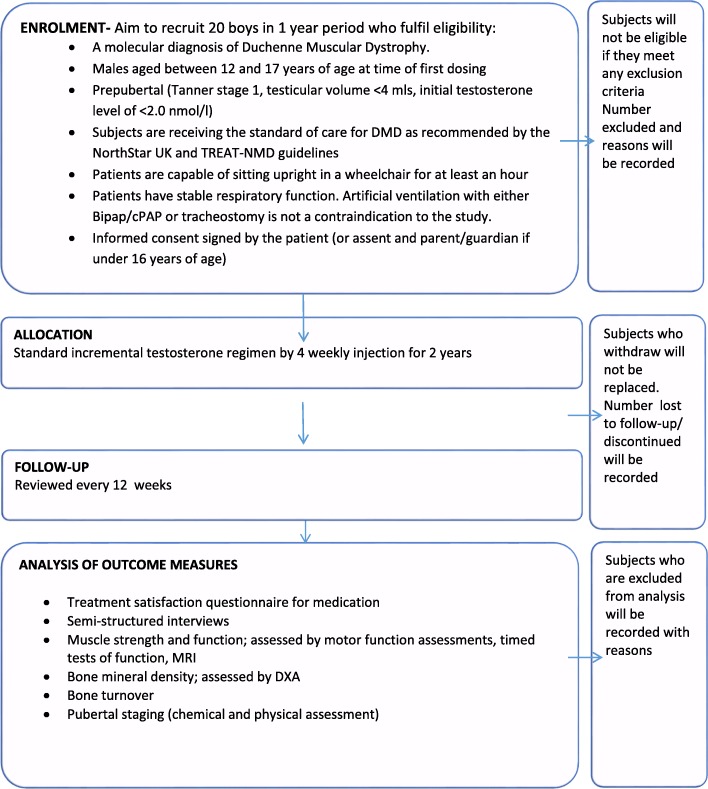
Testosterone study flowchart

Once the baseline visit is completed and he has received his first testosterone injection, every subject will be asked to return to either our centre or his local doctor every 4 weeks for further injections, see also Fig. 1. Every 24–28 weeks, he will also have a follow-up visit at the same time. The evaluations in this study will be carried out as part of standard care for Duchenne Muscular Dystrophy but will also be part of the study protocol and follow-up. Both medical care and research will be carried out at the same visit.

At each 4-weekly visit, the dosage of the testosterone may be increased depending on the regimen. This is the normal way to give testosterone to boys with pubertal delay, to try and match what the body would normally do, as closely as possible. If a subject experiences side effects due to the study drug, this will be documented and the study doctor may decide to interrupt or reduce the testosterone dose that he is receiving. Where possible, testosterone injections will also be given at study visits, to minimise inconvenience to participants.

### 12 weekly follow-up visits at 12, 40, 64 and 92 weeks: (at CRF or with local doctor)

- Testosterone level
- Pulse and blood pressure, skin examination
- Concomitant medications and adverse events recorded

### 24–28 weekly follow-up visits at 24–28, 52, 76–80, and 104 weeks (at CRF/great north children’s hospital)

- Blood and urine samples
- Pulse and blood pressure
- Concomitant medications and adverse events recorded
- General and pubertal examination
- Same physiotherapy assessment and breathing tests as at baseline
- Quality of Life questionnaire completion
- Treatment Satisfaction for Medication Questionnaire

### 52-week follow-up visit and 104 week visit (at CRF/MRI centre)

In addition to those at 24–28 weeks, the following tests will also be carried out:

- Muscle MRI
- Blood sample for routine laboratory tests, Vitamin D levels and bone markers
- Urine sample collection for analysis of bone markers
- Full-body DEXA scan (x-ray) for bone and muscle mass
- Echocardiogram and electrocardiogram

### At the 104-week follow-up only, in addition to the above

- X-ray of wrist
- Semi-structured questionnaire

### 116-week final visit

Final blood test to check testosterone level, 3 months after last injection.

Data collection will comprise:

#### Demographic data

Information will be collected regarding past medical history, social history, family history (including family history of pubertal delay), concomitant medications, allergies and hospital admissions.

#### Treatment satisfaction questionnaire for medication (TSQM)

The quantitative primary outcome measure will be a patient reported questionnaire validated to assess subject/parent global satisfaction with treatment, known as the Treatment Satisfaction Questionnaire for Medication (TSQM), version 1.4 The TSQM is a self-administered questionnaire that will be used at baseline and every 6 months thereafter. It is a 14-item instrument, yielding four subscale scores: global satisfaction, effectiveness, adverse events and convenience and has been validated for adults with chronic disease [25]. The total score from this will be the primary outcome measure to assess tolerability of testosterone given by intramuscular injection for the induction of pubertal delay. Self-report questionnaires are vital as there is a documented poor correlation between the perceived health related quality of life of adolescents with DMD and the views of their parents. Data has shown that parents grossly underestimate perception of son’s happiness and of perceived support from social networks [26]. The participants will complete questionnaires, with help from their parents if required.

#### Semi-structured interviews

The semi-structured interviews will be recorded and used to allow triangulation of the data obtained from the quantitative components and gain greater insight into the subjects’ views on the use of testosterone therapy. A topic guide will be used, but the individual interviews will be guided by the responses. In particular, we hope to explore the boy’s ideas, concerns and expectations prior to testosterone therapy and discover whether these have been met by their trial regimen. Experienced and suitably trained members of the study team will carry out the interviews. Coding and thematic analysis will be used. Semi-structured interviews were felt to be the most suitable format as several sensitive and potentially embarrassing issues are likely to be explored, which would not be suitable for discussion in a group setting. A semi-structured approach will be used to allow the boy’s the freedom to steer the conversation whilst still obtaining specific information regarding the boy’s attitude to testosterone therapy.

#### Vital signs

Vital signs will be recorded at screening, baseline and every subsequent visit to the CRF. These will consist of height, weight, BMI (calculated), blood pressure and pulse rate (beats per minute).

Standing height will be measured using a recently calibrated stadiometer. Subjects who are unable to stand with heels flat in an upright position due to Achilles tendon contractures will be measured with both standing height and ulna length. If the subject loses standing ability over the duration of the trial, ulna length only will be measured.

#### Functional motor assessments

Physiotherapy assessments will be carried out at 6 monthly intervals, as in routine clinical practice. The Performance of the Upper Limb (PUL 1.3) for DMD will be performed on all subjects. This is a measure of upper limb motor performance and its development and reliability have been reported [27]. North Star Ambulatory Assessment (NSAA) will also be used on the ambulant DMD subjects. It is a robust and well validated measure of ambulatory motor performance [28] which reflects activities of daily living such as walking, standing up from a chair, climbing a step, hopping, running and jumping. If a child loses the ability to walk independently during the study period, then only PUL will be used.

#### Timed and graded functional tests

Validated timed tests for the assessment of DMD will be performed. These comprise:

1. Rise from floor (within NSAA)
2. 10 m walk/run (within NSAA)
3. Stairs climb and descend
4. 6-min walk test

#### Assessments of muscle strength and range of movement

Standard assessments of muscle strength, function and range of movement will be performed by trained, highly experienced physiotherapists:

1. Knee flexion and extension
2. Elbow flexion and extension
3. Shoulder abduction
4. Grip strength
5. Assessment of workable reach space using Kinect

#### Spirometry (FVC, FEV1, peak cough flow)

Spirometry will be used to assess lung function and strength of respiratory function. If the patient is on non-invasive ventilation, the home ventilation team will also review them. Spirometry is routinely performed 6-monthly, during outpatient clinic appointments. This data will be collected to ensure that testosterone does not have an adverse effect on respiratory function.

#### Dual X-ray absorptiometry (DXA)

A DXA scan will be performed yearly to assess lean versus total body mass and to enable calculation of bone mineral density, adjusted for age, size and gender. Adjusted z-scores of lumbar spine and total body (minus head) will be recorded. This is routine clinical care in DMD patients who are receiving GC therapy.

#### Echocardiogram (echo) and electrocardiograph (ECG)

An echo and ECG will be carried out as standard practise at yearly intervals and reviewed by a consultant paediatric cardiologist. This data will be collected to ensure that testosterone does not have an adverse effect on cardiac function.

#### Laboratory assessments

As part of routine care within the endocrine assessment for delayed puberty, the following assessments will also be carried out at baseline and 12 weekly intervals:

- Testosterone, luteinizing hormone, follicle-stimulating hormone.

Additional blood and urine tests will be carried out at baseline and at 6-monthly intervals:

- Blood tests

1. Full blood count with differential, ferritin, urea and electrolytes, thyroid function tests, liver function tests, creatine kinase, calcium, magnesium, parathyroid hormone, 25-OHD, total and differential cholesterol levels, triglycerides, fasting glucose and insulin.
2. Prolactin, growth hormone, insulin-like growth factor-1, cortisol, ACTH, leptin, bone-specific alkaline phosphatase, beta crosslaps, osteocalcin, P1NP, RANKL, osteoprotegrin, sclerostin. These are optional bloods, which will be taken if the patient consents, from the same blood draw as the routine haematology and biochemistry bloods. The option to take an additional sample of blood for storage in the BioBank will also be offered.

- Urine calcium/creatinine ratio

#### Pubertal staging

- Bone age will be assessed at baseline and at study endpoint, using an Xray of the left wrist and the ‘Greulich and Pyle’ method [29].
- Testicular size will be assessed using a Prader orchidometer.
- Pubertal staging will be assessed using the validated Tanner Staging criteria [30]. If the subject refuses to allow the assessor to undertake pubertal staging, the Tanner criteria will be offered on a chart for self-validation.

#### PedsQL self-report questionnaire

Quality of life will be measured using the PedsQL questionnaire [31]. This will be administered at baseline and then at 6-monthly intervals to the subject themselves and also to their parent/guardian. The Generic Core Scale will be completed first and then the Neuromuscular Module.

#### Muscle MRI

Muscle MRI of the upper and lower limbs will be carried out at baseline, 52 weeks of follow-up and at the end of the study period. Scans will be carried out according to a standard protocol (see Additional file 1). If patients are unable to comply, their data will still be collected for other components of the trial.

### Statistical methods

#### Analysis of the primary outcome measure

This is an observational study designed to investigate the effect of testosterone on boys with DMD for the first time. The outcome measures have been chosen according to their clinical validity and importance on patient-reported, rather than parent-reported, measures. A 2-year follow up period has been chosen, as this is a practical time frame in which testosterone replacement therapy would usually act to trigger the natural pubertal process.

There are no previous studies and background data is limited hence formal power calculations are not possible. As such, formal hypothesis testing will not be carried out due to a probable lack of power. Statistical analyses will be based on descriptive statistics. Quality of life scores will be calculated according to validated scoring systems accounting for missing items.

Analysis will be performed on an intention-to-treat basis, using STATA version 13.0 [32]. Per protocol analyses may be carried out to inform future trial design.

#### Analysis of secondary outcome measures

Exploratory regression models will be used to investigate and quantify the relationship between testosterone levels at 104 weeks and important pre-specified clinical predictors and treatment, whilst accounting for stratification factors at randomisation. Results will be presented descriptively as parameter estimates (with associated confidence intervals).

Data from the semi-structured interviews will be analysed thematically. Two experienced qualitative researchers will code and analyse the emergent themes, to enable triangulation of data.

#### Interim analyses and criteria for the premature termination of the trial

The trial may be prematurely discontinued on the basis of new safety information, or for other reasons given by the Data Monitoring Committee and/or Trial Steering Committee, Sponsor, regulatory authority or ethics committee concerned.

### Statistical size calculations

We initially chose to recruit 20 boys with DMD as this represented a realistic recruitment target from the local population of boys with DMD and pubertal delay who are not already enrolled in a clinical trial. The target recruitment figure of 20 was chosen pragmatically based upon what was considered feasible and realistic given the rare nature of the condition and the expected availability of eligible patients within the recruitment period and the recruitment area. As such formal statistical power calculations are not possible. This was discussed and agreed by the statistician within the Clinical Trials Unit. The availability of eligible participants within the said timeframe proved to be lower than anticipated, with the final recruitment achieved at 15 participants. This final recruitment figure was discussed within the Steering Committee where it was confirmed that this downward revision has no impact upon the data analysis in view of the original target not being statistically powered and that no formal hypothesis testing is planned due to this lack of power. The amended sample size was submitted to the Ethics Committee and approved as a minor amendment.

As an observational study, the aim of this trial is to provide the evidence of efficacy and acceptability of testosterone replacement therapy, on which to enable power calculations for a larger multi-centre trial.

The decision to continue with further research would be based on:

Evidence of acceptability and clinical effectiveness of testosterone treatment according to:

i) Feedback in qualitative interviews
ii) Scores from the Treatment Satisfaction for Medication Questionnaires
iii) Changes in secondary outcome measures

### Data monitoring and reporting

Adverse events and concomitant medications will be assessed and documented at baseline and at all of the subsequent trial visits. A structured record of adverse events and reactions will be made in the database so that tolerability can be accurately assessed by the trial team.

A trial management group, trial steering committee and data monitoring committee have been set up and will oversee the trial and all associated regulatory matters. A trial monitoring plan has been developed and agreed by the trial management group, trial steering committee and sponsor. On-site monitoring visits will be conducted in accordance with the monitoring plan. The data monitoring committee is independent from the sponsor and does not have any competing interests.

The trial will be conducted in accordance with the Medicines for Human Use (Clinical Trials) Regulations 2004 and subsequent amendments. All parties must abide by these regulations and the ICH GCP guidelines.

Clinical Trial Authorisation was obtained from the MHRA prior to the start of the trial and the trial Sponsor will notify the MHRA of any substantial amendments that require review. These substantial amendments will not be implemented until the MHRA have issued an acceptance of the amendment. The Development Safety Update Report will be submitted to the MHRA by the CI on an annual basis until the end of the trial.

The procedures are compliant with the Ionising Radiation (Medical Exposure) Regulations and appropriate review by a Medical Physics Expert and Clinical Radiation Expert has been undertaken.

### Withdrawal criteria

Participants have the right to withdraw from the trial at any time without having to give a reason. Investigator sites should try to ascertain the reason for withdrawal and document this reason within the Case Report Form and participant’s medical notes. The investigator also has the right to withdraw patients from the study drug in the event of inter-current illness, side effects, serious adverse events, suspected unexpected serious adverse reactions, administrative reasons or other reasons. Should a patient stop taking testosterone through their own choice, efforts will be made to continue to obtain follow-up data, with the permission of the patient.

The Investigator may discontinue a participant from the trial at any time if the Investigator considers it necessary for any reason including:

- Symptomatic deterioration
- Unacceptable toxicity
- Participant withdrawal of consent
- Significant protocol deviation or non-compliance
- Investigator’s discretion that it is in the best interest of the participant to withdraw
- An adverse event that requires discontinuation of the trial medication or renders the participant unable to continue in the trial
- Termination of the clinical trial by the sponsor

Participants who withdraw from the trial will not be replaced.

#### Participant timeline

The study opened for recruitment in December 2015 and the first patient was consented in the same month. The study is now closed for recruitment (closed December 2016) as 15 participants were recruited, which is consistent with the amended target and the patients are currently completing the 27 month study period. The last patient will complete their last study visit in February 2019. No data from the study has been analysed yet. Discussions are currently underway to seek funding to run an observational extension study, enabling the same cohort of patients to be followed up for a further two years.

### Data handling

Data collected on paper Case Report Forms will be entered onto a secure validated clinical data management system. A unique study identifier will be used to identify participants on case report forms. Data will be handled, computerised and stored in accordance with the Data Protection Act 1998. No participant identifiable data will leave the study site. Paper copies of all study related laboratory results will be annotated, signed and dated and filed in the medical notes/records. The quality and retention of study data will be the responsibility of the Chief Investigator. All study data will be retained in accordance with the latest Directive on GCP (2005/28/EC) and local policy.

Clinical information will not be released without the written permission of the participant, except as necessary for monitoring and auditing by the Sponsor, its designee, Regulatory Authorities, the Data Monitoring Committee (DMC) or the REC. Secure anonymised electronic data may however be released to the Study Statistician for analysis. The PI and study site staff involved with this study may not disclose or use for any purpose other than performance of the study, any data, record, or other unpublished, confidential information disclosed to those individuals for the purpose of the study. Prior written agreement from the Sponsor or its designee must be obtained for the disclosure of any said confidential information to other parties.

### Protocol version and amendments

This study protocol is based on version 1.2 of the trial protocol. The first minor amendment was made to address the change in recruitment target from 20 participants to 15. The second minor amendment reflected a small change in the SmPC.

### Dissemination

It is planned to publish this study in peer review articles and to present data at national and international meetings. Results of the study will also be reported to the Sponsor and Funder, and will be available on their web site. The Trial Steering Committee and Funder will review all manuscripts, abstracts or other modes of presentation prior to submission. Individuals will not be identified from any study report. Participants will be informed about their treatment and their contribution to the study at the end of the study, including a lay summary of the results.

## Additional file

Additional file 1: MRI Standard Operating Procedure for Testosterone in DMD study. (DOCX 104 kb)

## Abbreviations

CI: Chief investigator
CRF: Clinical research facility
DMD: Duchenne muscular dystrophy.
DXA: Dual x-ray absorptiometry
GC: Glucocorticoid
GH: Growth hormone
ICH GCP: International Conference on Harmonisation Good Clinical Practice
MHRA: Medicines for Health Regulatory Authority
MRI: Magnetic resonance imaging
NSAA: Northstar ambulatory assessment
REC: Research ethics committee
SmPC: Summary of product characteristics
