# Supplementary material for: Observational study of clinical outcomes for testosterone treatment of pubertal delay in Duchenne muscular dystrophy
Source: BMC Pediatr. 2019 Apr 25;19:131. doi: 10.1186/s12887-019-1503-x (PMC6482579; doi:10.1186/s12887-019-1503-x)
Supplement: Supplementary file 1 — MRI Standard Operating Procedure for Testosterone in DMD study. (DOCX 104 kb) [file 12887_2019_1503_MOESM1_ESM.docx]

## MRI Standard Operating Procedure for Testosterone in DMD study

Participants will be examined using a 3.0T Philips MRI scanner, based at the Newcastle Magnetic Resonance Centre at the Campus for Ageing and Vitality, Newcastle University. The examination will avoid the movement of the patient off the scanner bed after the initial positioning.

Patients will be required to lie supine on the scanner bed. Arrangements will be made to transfer those with mobility difficulties either using a patient hoist, or through the use of an MR-compatible wheelchair as appropriate.

The protocol will assess the muscle volume and fat fraction of the lower and upper limbs on the patients’ anatomical left side as follows:

**(i) Lower limb examination**

The fat fraction of the lower limb will be performed using a 3-point Dixon sequence (3D, TR=10ms, FA=3degrees, TE=4.4,5.18,5.96ms, 48 slices), which will be accelerated using compressed sensing. These scans have a duration of 1 minute to cover the lower leg and 1 minute for the upper leg. For consistency of anatomical examination we will landmark the prescription with respect to the long bones, with measurement to allow consistent placement, allowing for growth. The scans will permit the estimation of muscle fat fraction and muscle volume.

**(ii) Upper limb examination**

The upper limb examinations will be performed using a local receiver surface coil for optimal sensitivity. The upper limb will be examined in a relaxed position by the patient’s side. To obtain the best magnet homogeneity, the patient will be moved over to the side of the magnet, with the upper limb not being scanned against the side of the magnet.

We will perform T1 weighted imaging of the lower and upper arm to allow estimation of muscle volume, and 3-point Dixon imaging to permit the quantification of muscle fat replacement. Each Dixon scan will have a duration of 6 minutes. Both the lower and upper sections of the arm will be studied.

T2 measurement of the lower arm will be made, using a turbo spin echo sequence without fat suppression, duration 8 minutes.

**Analysis**

The 3-point Dixon scans will be analysed off-line to produce fat-fraction maps and analysed by region of interest analysis, to determine the percentage of fat replacement. T2 relaxation times for the muscles of the arm will be calculated. Images at the different time points will be registered before analysis.
